# Impact of Tenofovir Alafenamide Sub‐Dermal Implant Insertion Site Scarring on Acceptability and HIV Prevention Preferences: A Prospective Cohort Study in Durban, South Africa

**DOI:** 10.1002/jia2.70107

**Published:** 2026-04-16

**Authors:** Tanuja N. Gengiah, Lara Lewis, Ishana Harkoo, Nqobile Myeni, Leila E. Mansoor, Salim S. Abdool Karim, Quarraisha Abdool Karim

**Affiliations:** ^1^ Centre for the AIDS Programme of Research in South Africa University of KwaZulu‐Natal Durban South Africa; ^2^ Department of Epidemiology Columbia University Mailman School of Public Health New York City New York USA

**Keywords:** acceptability, implant site reactions, implant, PrEP preferences, scarring, TAF

## Abstract

**Introduction:**

The CAPRISA 018 Phase I trial evaluated the safety, tolerability and pharmacokinetics of a 110 mg tenofovir alafenamide (TAF) implant for HIV prevention in South African women. This follow‐up cohort study, CAPRISA 097, assessed the long‐term resolution of implant site reactions (ISRs) after implant removal and explored user acceptability and implant attribute preferences to inform the development of next‐generation pre‐exposure prophylaxis (PrEP) implants.

**Methods:**

Women previously enrolled in CAPRISA 018 were recruited and followed quarterly for 12 months between 13 October 2022 and 27 October 2023. ISR prevalence, severity and resolution were evaluated at each visit. Implant acceptability, implant attribute preferences and PrEP preferences were assessed at enrolment and at month 12.

**Results:**

Of 36 eligible participants, 35 were enrolled a median of 299 days after implant removal (IQR: 243–490). At enrolment, all 35 participants (100%) had ongoing mild (Grade 1) scarring, with additional findings of hyperpigmentation (14%), induration (6%) and hypopigmentation (3%). By study exit, scarring persisted in all participants (median duration: 623 days; IQR: 579–819), while hyperpigmentation and induration remained in two and one participant, respectively. Acceptability ratings for implant visibility were similar at enrolment and month 12 (77.1% vs. 75.0%), as were ratings for pain (68.6% vs. 78.1%). Side effects due to ISRs received the highest “very unacceptable” ratings, in 37.1% of participants at enrolment and 21.9% at study exit. Scarring was considered acceptable by 65.7% of participants at enrolment, increasing to 78.1% at exit. Perceived partner interest in the various PrEP products aligned with participant interest. A palpable 12‐month implant was acceptable to most participants, whereas increased length, width or stiffness reduced the likelihood of use. Preferred PrEP options were a 12‐monthly implant (38.2% at enrolment vs. 50.0% at month 12), a 6‐monthly injection (29.0% vs. 37.5%) and daily oral PrEP tablets (12.0% vs. 3.0%).

**Conclusions:**

Mild but persistent scarring was observed following TAF implant removal, with limited cases of hyperpigmentation and induration. Despite these local side effects, a 12‐monthly implant remained the most preferred PrEP option among women previously enrolled in the TAF implant trial.

## 1 Introduction

Global HIV transmission has declined since 2010, yet annual incidence remains high [1, 2]. In eastern and southern Africa, adolescent girls and young women (AGYW) account for 26% of all new cases, are at three‐fold higher risk than male peers [1], often acquiring HIV 3−5 years earlier [3], underscoring the need for pre‐exposure prophylaxis (PrEP) as a priority intervention for AGYW.

HIV PrEP options include oral tenofovir‐containing PrEP [4, 5, 6, 7, 8] and, more recently, long‐acting (LA) antiretroviral drugs formulated as dapivirine intravaginal rings (IVRs) [9] or injectables like cabotegravir [10] and lenacapavir [11], which are highly effective for AGYW. While daily oral PrEP is consistently effective in men who have sex with men and transgender women [4, 12], trial results have been inconsistent in women, due to adherence challenges [5, 6, 7, 8] and biological factors [13, 14]. Women require 4−6 doses of oral PrEP a week to be protected [15], with suboptimal uptake and low persistence being reported [16]. Although the South African Health Products Regulatory Authority has registered the dapivirine IVR and cabotegravir LA [17], access remains limited outside of demonstration projects.

IVRs and injectables offer advantages over oral PrEP, but challenges remain that sustained‐release sub‐dermal implants may overcome. The high potency of tenofovir alafenamide (TAF) makes an annual sub‐dermal PrEP implant feasible [18, 19]. The challenge, however, is the risk for local reactions demonstrated in preclinical models [20, 21]. The CAPRISA 018 trial, the first‐in‐human evaluation of a sustained‐release TAF implant, found acceptable implant characteristics but intolerable local reactions leading to early removals [22, 23]. This follow‐up CAPRISA 097 cohort study assessed long‐term sequelae post‐removal and explored acceptability, preferred product attributes and PrEP choices, providing user‐centred insights to guide future PrEP implant development.

## 2 Methods

### 2.1 Study Participants and Design

The CAPRISA 018 Phase I trial enrolled across two groups. Group 1 (*n* = 6) received a single 110 mg TAF implant for 28 days, which was removed at day 28, with study exit at week 8. Group 2 (*n* = 30) participants were randomized to receive one or two implants in a 4:1 active‐to‐placebo ratio and were followed through week 48, when implants were removed, with study exit at week 52 [22, 24]. Some Group 2 participants had implants removed early (*n* = 11). The 40 mm long TAF silicone implant was inserted into the bicep region of the non‐dominant arm, using trocar‐guided insertion. Trial findings have been published elsewhere [22, 23].

This prospective cohort study enrolled CAPRISA 018 participants after implant removal. Study visits occurred quarterly and ended at month 12. This study was conducted at a single site in Durban, South Africa, from 13 October 2022 to 27 October 2023. All study participants provided written informed consent, and ethics approval was obtained from the University of KwaZulu‐Natal Biomedical Research Ethics Committee (BREC/00004541/2022)

### 2.2 Implant Site Reaction Assessments

At each visit, a trained study clinician performed a standardized assessment of the implant site for implant site reactions (ISRs) unresolved at CAPRISA 018 exit or had developed subsequently. Assessments included evaluation for erythema, induration, swelling, pain, tenderness, bruising, hyperpigmentation, hypopigmentation, pruritus and visible scarring. The maximum diameter (cm) of any reaction was measured, and (where applicable), surface area (cm^2^) was estimated by multiplying the longest diameter by its perpendicular width.

ISR severity was graded using the DAIDS Table for Grading the Severity of Adult and Pediatric Adverse Events, Version 2.1 (March 2017), applying criteria for injection and infusion site reactions. ISRs <2.5 cm in diameter (<6.25 cm^2^ surface area), usually below grading thresholds, were conservatively graded similarly to reactions between 2.5 and <5 cm (6.25−25 cm^2^) as Grade 1; ≥5 to <10 cm (≥25 to <100 cm^2^) as Grade 2; and ≥10 cm (≥100 cm^2^) as Grade 3. Participant‐reported symptoms (e.g. pain, itching, tenderness) were documented and incorporated into grading where applicable. Scarring was graded according to the “Estimating severity grade for parameters not identified in the grading table” row.

Resolution was defined as the complete absence of visible or palpable abnormality at the insertion site. Persistent ISRs were defined as any ongoing clinical finding present at study exit. Scarring was defined as visible fibrotic skin change at the insertion site after resolution of acute inflammatory signs and was documented descriptively (flat vs. raised) and skin colour changes as hyperpigmented versus hypopigmented.

### 2.3 Post Removal Implant Acceptability

Implant acceptability was assessed at enrolment, month 6 and month 12 (study exit). Only entry and exit data are reported, as month 6 provided no additional insights.

### 2.4 Implant Product Attribute Preferences and HIV PrEP Method Preferences

TAF implant attribute surveys evaluated preferences for potential implant modifications. PrEP preference surveys assessed participant and perceived partner preferences for implants versus oral pills, injectables, vaginal rings or vaginal inserts. Both assessments were conducted at study entry and exit.

### 2.5 Statistical Considerations

The median time from implant removal to complete ISR healing was calculated in days. When the exact healing date was unknown, the midpoint between the last visit with ISRs and the first visit without ISRs was used. Participants with unresolved ISRs at study exit were censored at their last visit. The proportion of participants with ISRs at study end, overall and by ISR type, was calculated using 95% Wilson score confidence intervals. For implant acceptability, attribute preferences and PrEP method preferences, categorical variables were summarized as frequencies and percentages, and continuous variables as medians with interquartile ranges (IQRs).

All statistical analyses were conducted in SAS version 9·4 (SAS Institute Inc., Cary, NC, USA).

## 3 Results

Of the 36 women previously enrolled in the CAPRISA 018 implant trial, 35 consented to enrolment. The median (IQR) age of participants was 29 (23−32) years, all were Black African and five participants (14%) had prior contraceptive implant use experience. Two participants stopped participation early, 33 completed the study and had a median (IQR) follow‐up of 336 (337−366) days.

The mean (range) number of ISRs per participant present at implant removal in the parent CAPRISA 018 trial was 4 (1−9), and 92% were mild (Table 1). Hyperpigmentation was more frequent in active implant users (79%) compared to placebo implant users (17%). A median of 299 (IQR: 243–490) days after implant removal, the mean number of ISRs per participant was 1 (range: 1–3), and all ISRs were mild. Scarring was present in all participants, and hyperpigmentation in five participants.

**TABLE 1:** ISR prevalence, severity and duration.

	Implant removal in CAPRISA 018				CAPRISA 097 enrolment				CAPRISA 097 12‐month visit				
ISR type	*n*/*N* (%)^a^	% Grade 1^b^	% Grade 2^b^	% Grade 3^b^	*n*/*N* (%)^a^	% Grade 1^b^	% Grade 2^b^	% Grade 3^b^	*n*/*N* (%)^a^	% Grade 1^b^	% Grade 2^b^	% Grade 3^b^	Median (IQR) days to resolution from implant removal^c^
***All participants***													
Hyperpigmentation	24/35 (69)	96	4	.	5/35 (14)	100	.	.	1/32 (3)	100	.	.	141 (42−295)
Hypopigmentation	2/35 (6)	100	.	.	1/35 (3)	100	.	.	0/32 (0)	.	.	.	178 (72−284)
Induration	29/35 (83)	90	10	.	2/35 (6)	100	.	.	1/32 (3)	100	.	.	57 (24−123)
Scarring	35/35 (100)	97	3	.	35/35 (100)	100	.	.	32/32 (100)	100	.	.	623 (579−819)
Tenderness	17/35 (49)	100	.	.	0/35 (0)	.	.	.	0/32 (0)	.	.	.	9 (3−20)
Other^d^	25/35 (71)	76	16	8	0/35 (0)	.	.	.	0/32 (0)	.	.	.	24 (7−30)
***Participants with active implants***													
Hyperpigmentation	23/29 (79)	96	4	.	5/29 (17)	100	.	.	1/27 (4)	100	.	.	151 (37−326)
Hypopigmentation	2/29 (7)	100	.	.	1/29 (3)	100	.	.	0/27 (0)	.	.	.	178 (72−284)
Induration	27/29 (93)	89	11	.	2/29 (7)	100	.	.	1/27 (4)	100	.	.	63 (23−151)
Scarring	29/29 (100)	97	3	.	29/29 (100)	100	.	.	27/27 (100)	100	.	.	675 (606−848)
Tenderness	14/29 (48)	100	.	.	0/29 (0)	.	.	.	0/27 (0)	.	.	.	3 (2−20)
Other^d^	22/29 (76)	73	18	9	0/29 (0)	.	.	.	0/27 (0)	.	.	.	25 (7−32)
***Participants with placebo implants***													
Hyperpigmentation	1/6 (17)	100	.	.	0/6 (0)	.	.	.	0/5 (0)	.	.	.	79 (79−79)
Hypopigmentation	0/6 (0)	.	.	.	0/6 (0)	.	.	.	0/5 (0)	.	.	.	.
Induration	2/6 (33)	100	.	.	0/6 (0)	.	.	.	0/5 (0)	.	.	.	54 (52−56)
Scarring	6/6 (100)	100	.	.	6/6 (100)	100	.	.	5/5 (100)	100	.	.	553 (537−567)
Tenderness	3/6 (50)	100	.	.	0/6 (0)	.	.	.	0/5 (0)	.	.	.	16 (9−28)
Other^d^	3/6 (50)	100	.	.	0/6 (0)	.	.	.	0/5 (0)	.	.	.	7 (6−28)

^a^ Number of participants with ISR present over the total number of participants examined.

^b^ Worst severity grading used per ISR type. Grade 1 = mild, 2‐moderate, 3‐severe.

^c^ Unresolved ISRs were censored at the last visit date.

^d^ Includes administration site discomfort, application site excoriation, implant site abscess, implant site bruising, implant site erythema, implant site oedema, implant site pain, implant site pruritus, implant site pustules and implant site ulcer.

Post‐implant removal, scarring persisted a median of 623 (IQR: 579–819) days overall; 675 (IQR: 606–848) and 553 (IQR: 537–567) days in those who had received active and placebo implants, respectively.

**FIGURE 1:**
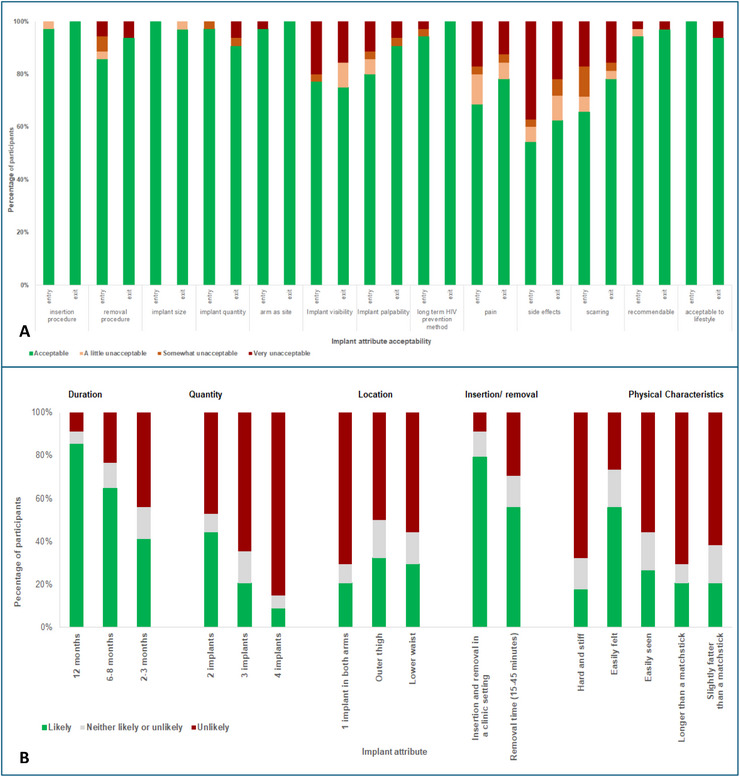
Panel A: Acceptability of implant attributes, insertion/removal procedures at study entry and exit (month 12). Panel B: Likelihood of using implant(s) with given attribute.

At enrolment and 12‐month exit (Figure 1, panel A), ≥80% of participants found the implant's size, number, insertion/removal procedures, arm as insertion site, palpability, HIV prevention potential, lifestyle compatibility and willingness to recommend to others as acceptable. Acceptability for implant visibility was 77.1% versus 75.0%, and for pain, 68.6% versus 78.1%, respectively. ISRs had the highest “very unacceptable” ratings (37.1% at entry vs. 21.9% at exit). Scarring persisted in all cases but was considered acceptable by 78.1% of participants at exit.

Participants’ likelihood of future implant use was assessed based on product attributes (Figure 1, panel B). A 12‐month implant that was palpable was likely to be used by most participants, while increasing the length, width or silicone stiffness would reduce the likelihood of use. The outer thigh was considered by 32% of participants as an alternate anatomical location for implantation.

Table 2 summarizes which PrEP modalities participants prefer, alongside the modalities they believe their partners would prefer. Injectable LA PrEP and the implantable PrEP generate the most interest. At enrolment and month 12, the preferred PrEP options were a 12‐monthly implant (38.2% vs. 50%), a 6‐monthly injectable option (29% vs. 37.5%), oral (11.8% vs. 6.3%) or vaginal PrEP tablets (11.8% vs. 0%), a 6‐monthly implant (0% vs. 3.1%) and the vaginal ring (2.9% vs. 0%).

**TABLE 2:** PrEP user and perceived reported partner preferences at enrolment and 12‐month visit.

PrEP option	*n* (%) interested in product		*n* (%) perceived partner interest in product^a^		*n* (%) product as preferred choice^b^	
	Enrolment (*n* = 34)	12 months (*n* = 32)	Enrolment (*n* = 31)	12 months (*n* = 27)	Enrolment (*n* = 34)	12 months (*n* = 32)
2‐monthly long‐acting injection	25 (74)	17 (53)	18 (58)	11 (41)	0 (0)	0 (0)
6‐monthly long‐acting injection	27 (79)	27 (84)	20 (65)	22 (81)	10 (29)	12 (38)
Daily oral pill	16 (47)	13 (41)	11 (35)	9 (33)	4 (12)	1 (3)
Oral pill before and after sex	15 (44)	14 (44)	10 (32)	11 (41)	0 (0)	2 (6)
6‐monthly implant	17 (50)	19 (59)	19 (61)	15 (56)	0 (0)	1 (3)
12‐monthly implant	25 (74)	25 (78)	22 (71)	21 (78)	13 (38)	16 (50)
Monthly vaginal ring	5 (15)	4 (13)	4 (13)	3 (11)	1 (3)	0 (0)
2‐monthly vaginal ring	6 (18)	6 (19)	5 (16)	3 (11)	1 (3)	0 (0)
Vaginal tablet	12 (35)	7 (22)	10 (32)	6 (22)	4 (12)	0 (0)

^a^ Three and five participants did not have partners at enrolment and 12 months, respectively.

^b^ One participant did not complete the user preference survey at enrolment and 12 months, respectively.

## 4 Discussion

This study assessed long‐term ISR resolution, TAF implant attribute preferences and acceptability. A median of 9.8 months post‐implant removal, all women had insertion site scarring and reported between 1−3 ongoing ISRs. During follow‐up, scarring persisted in all participants. While implant size and integration into daily life were viewed positively, ISRs affecting appearance reduced overall acceptability. Despite the persistent scarring, most participants maintained a preference for the 12‐monthly implant compared to other PrEP options.

As described in the TAF implant trial [22], mild to moderate ISRs have also been reported in trials of other LA PrEP modalities. Most notably, the two Phase I islatravir implant trials with implants inserted for 12 weeks [25, 26], ISR resolution post‐implant removal took approximately 3.5 months in one study [25], and 2−3 months in the other, mostly for ISRs like erythema and induration [26]. Other subcutaneously administered PrEP, like CAB400D injection, was associated with more frequent injection site swelling, induration, erythema and nodules compared to intramuscular administration, lasting several months [27]. In the PURPOSE 1 trial, mild/moderate subcutaneous injection site nodule formation with lenacapavir occurred in 68% of participants but dissipated with subsequent dosing [11]. In contrast, in our study, mild scarring and pigmentation persisted for several months post‐removal, with scarring still evident almost 2 years after removal. Comparatively, long‐term contraceptive implants (synthetic hormones) demonstrate better local tolerability (<1% ISR rate) than the TAF implant [28]. Subcutaneous tissue reactions that lead to long‐term sequelae at the implant insertion site impact both tolerability and acceptability and are a key consideration for future implant candidates.

Although a 12‐month implant was preferred for adherence benefits, its long‐term success depends on tolerability and user experience. Scarring, hyperpigmentation and visibility, though medically mild, were socially and psychologically significant, reducing acceptability and driving higher‐than‐expected early removals [23]. Despite persistent ISRs, most participants still favoured a 12‐monthly implant over injectables, oral or other PrEP options. This reinforces the value of LA PrEP choice for women with adherence challenges and indicates a willingness to accept some degree of tolerability trade‐off in exchange for reduced dosing burden. Previous acceptability assessments of CAB‐LA have shown that African trial participants were less concerned with the physical experience and instead prioritized product attributes [29]. However, the acceptability of these trade‐offs may not extend to new users without prior PrEP implant experience. In discrete choice experiments, implants with a 12‐month duration that were biodegradable or did not require surgical removal were preferred, and the 6‐monthly injectable was favoured over the annual implant [30].

This study highlights several user preferences to guide key attributes for future implant design. These data suggest that similar or smaller implant sizes, comfortable device material, could improve tolerability, as also corroborated in previous research [31]. Unlike in studies which favoured flexible implants that could not be felt [32], palpability was a positive attribute, distinguishable from implant visibility associated with ISRs. This is likely because participants felt reassured by themselves physically confirming the implant remained in place, providing protection and had not migrated. In contexts where stigma is of concern, any visible sign of PrEP use may raise concerns about confidentiality and social acceptability [33]. For women, whose risk of acquiring HIV often intersects with gender‐based violence and unequal power dynamics in relationships [34], the ability to use prevention discreetly is critical. Persistent scarring or pigment changes could inadvertently reveal PrEP use and expose users to judgement, potentially undermining the benefits of an implant designed to reduce reliance on daily adherence. Lastly, consideration of alternative implantation sites in this study revealed that participants were open to locations such as the outer thigh, which may offer greater discretion. This provides more specific direction for future implant design compared to previous studies, where participants expressed interest in alternate sites but were unsure of their preference [30].

A strength of this study is its longitudinal design, with up to 20.5 months of post‐removal follow‐up time, enabling detailed assessment of ISR duration. Including participants with direct TAF implant experience grounds the findings in lived experience, making them valuable for next‐generation implant design. However, several limitations should be noted. First, the relatively small sample size limits generalizability. Second, the study relied on participant self‐reports, several months after implant removal, to assess aspects of acceptability and PrEP preferences, which may contribute to recall bias. Perceived partner preferences were subjective and of limited inferential value. Third, the cohort may not reflect the broader population of potential PrEP users, as all participants had previously consented to be part of a clinical trial and may have different thresholds for acceptability or willingness to try biomedical interventions. Despite these limitations, the study provides essential real‐world evidence on the long‐term tolerability of subdermal HIV prevention implants and contributes data to inform the user‐centred development of future PrEP technologies.

## 5 Conclusions

Participants’ direct experiences with the implant offer critical insights into tolerability, highlighting areas for improvement in product formulation and delivery. While the functional aspects of the implant were largely acceptable, cosmetic reactions at the insertion site significantly influenced participants’ attitudes and preferences. Addressing ISR‐related tolerability is essential to enhance acceptability and support the future research and development of PrEP implants.

## Author Contributions

TNG and QAK conceived and designed the study. The study was overseen by TNG and QAK. IH collected the clinical and safety data. NM and LEM provided study operational oversight. LL and TNG analysed the data. SSAK, TNG, LL and QAK interpreted the data. All authors reviewed the final version of this manuscript and consented to publication.

## Funding

The CAPRSA 097 study was funded by the European and Developing Countries Trial Partnership (EDCTP) (grant no: SRIA2015‐1061), as a project of the EDCTP2 programme supported by Horizon 2020, as well as the South African National Department of Health and the South African Medical Research Council (Project code 96151). In addition, the Department of Science and Innovation and the National Research Foundation contributed funding for this research through the DSI‐NRF Centre of Excellence in HIV Prevention (UID:96354).

## Conflicts of Interest

The authors declare no conflicts of interest.

## Data Availability

Study data sets will be made available to investigators whose proposed use of the data has been approved by the CAPRISA Scientific Review Committee. Requests to access the data can be made through the CAPRISA website (www.caprisa.org).
